# UPDATED PROTOCOL: Universal school‐based programmes for improving social and emotional outcomes in children aged 3–11 years: An evidence and gap map

**DOI:** 10.1002/cl2.1346

**Published:** 2023-08-04

**Authors:** Jennifer Hanratty, Sarah Miller, Leonor Rodriguez, Paul Connolly, Jennifer Roberts, Seaneen Sloan, Aoibheann Brennan‐Wilson, Daragh Bradshaw, Christopher Coughlan, Nicole Gleghorne, Laura Dunne, Sharon Millen, Aimee Smith, Cadhla O'Sullivan

**Affiliations:** ^1^ Centre for Effective Services Belfast UK; ^2^ Campbell UK & Ireland, School of Social Sciences Education and Social Work Queen's University Belfast Belfast Northern Ireland; ^3^ School of Social Sciences, Education and Social Work Queen's University Belfast Belfast Northern Ireland; ^4^ Faculty of Arts, Humanities and Social Sciences Ulster University Belfast Northern Ireland; ^5^ School of Education University College Dublin Dublin Ireland; ^6^ Centre for Evidence and Social Innovation Queen's University Belfast Belfast Northern Ireland; ^7^ Department of Psychology University of Limerick Limerick Republic of Ireland; ^8^ Campbell UK and Ireland Queen's University Belfast Belfast Northern Ireland; ^9^ UCD School of Education University College Dublin Belfield Republic of Ireland

## Abstract

This is the protocol for an evidence and gap map. The objective of this EGM is to identify and map all primary studies (including randomised and cluster randomised trials) and systematic reviews on universal, school‐based social and emotional learning programmes for young children (3–11 years) to create a live, searchable, and publicly available evidence and gap map.

## 1 BACKGROUND

This updated protocol for an evidence and gap map (EGM) replaces the original protocol for a systematic review of universal school‐based programmes for improving social and emotional outcomes in children aged 3–11 years. When the searches for the intended review were conducted in 2018, over 41,000 studies were identified for screening. The scale of work unfortunately exceeded the team's capacity and resource to complete it, meaning that the project came to a halt for a period of two years. During this time, EGMs have emerged as an exciting and informative way to synthesise a large body of research. Given the number of existing systematic reviews and trials in the area of social and emotional learning (SEL) an EGM was deemed the most sensible starting point to map the evidence first and then carry out more targeted reviews (in areas identified by the EGM as ripe for review) in the future.

The underlying premise of the proposed EGM is the same as the original review. Thus, the title remains unchanged, and all the original inclusion criteria (relating to types of study designs, participants, interventions, and outcomes) remain as described in the original protocol. The search strategy was implemented as originally designed.

### 1.1 The problem, condition or issue

Social and emotional competence is vital for forming and sustaining good relationships, solving everyday problems, adapting behaviour and making healthy choices throughout life. It includes self‐awareness, understanding and working with others, controlling emotions and caring about oneself and others. Through SEL, children and adults are able to develop the skills necessary to work and live effectively in society, yet a significant proportion of pupils struggle with one or more facets of social and emotional development (Doll et al., 2014; Jones & Bouffard, 2012; Zins, 2004). Estimates suggest that between 9.5% and 14.2% of children under five experience some form of social and emotional problem which negatively impact their functioning, development and school‐ readiness (Brauner & Stephens, 2006).

Underdeveloped social and emotional competencies can have consequences which are far reaching and long lasting (Daly et al., 2015; Durlak, 2015; Moffitt et al., 2011). Deficits in basic skills—such as the ability to identify emotions—can affect all stages of the lifespan, including being rejected by others; exclusion from peer activities; being victimised; and lower peer‐rated popularity (Lemerise & Arsenio, 2000; Leppanen & Hietanen, 2001; Mostow et al., 2002). Chronic physical aggression during primary school increases the risk of violence and delinquency through adolescence in boys (Broidy et al., 2003; Nagin & Tremblay, 1999), which in the long term can lead to destructive forms of emotion management, such as alcohol abuse. Poor self‐regulation in childhood is associated with negative outcomes in adulthood including poorer health, social and economic outcomes (Compas et al., 2004; Daly et al., 2015; Kubzansky et al., 2011; Moffitt et al., 2011), lack of (Nota et al., 2004) and increased criminal behaviour (Henry et al., 1999).

Additionally, research has identified that children who experience adverse childhood experiences are at a higher risk of developing lifelong, cumulative and adverse difficulties (Larkin et al., 2012). This can have a detrimental impact on their social, emotional and cognitive development, leading to lifelong somatic and mental health difficulties (Kovács‐Tóth et al., 2021). Therefore, specific groups of children may benefit from early recognition and risk reduction through SEL interventions. SEL programmes have the capacity to prevent multiple problems and support children who are repeatedly exposed to adverse experiences, particularly those from economically disadvantages minority backgrounds (Green et al., 2021). Cefai et al. (2018) consider that social and emotional education can be offered in schools to all children, including those who experience different forms of disadvantage.

SEL programmes aim to intervene early to address skill deficits and equip children with the social and emotional competencies they need for life (Eisenberg et al., 2010; Moffitt et al., 2011). Attention has increasingly turned to facilitating SEL in the classroom as a means to help children develop skills such as empathy, emotional regulation and behaviour management strategies, that will enable them to become functioning members of society in adulthood (Humphrey, 2013; Humphrey et al., 2010; Wigelsworth et al., 2022). Due to the increasing attention given to school‐based SEL interventions, the scope of this EGM will focus on these types of interventions, however, this excludes other types of existing SEL interventions for families and teacher trainings. This also restricts the age group included in this EGM to school aged children. Additionally, consensus exists regarding the crucial role of SEL in education, however, variation in evidence exits as well as understanding the meaning of whole school approaches (Wigelsworth et al., 2022). Research has found positive outcomes associated with SEL, however, some of these results have shown less positive results associates with inconsistent or ineffective implementation (Jones et al., 2018).

### 1.2 Scope of the EGM

This EGM will identify primary studies (randomised and cluster randomised trials) as well as systematic reviews on universal, school‐based SEL programmes for young children to create a live, searchable, and publicly available EGM.

The rows within the EGM matrix will represent different types of interventions, categorised according to the CASEL^1^ framework which identifies five interrelated areas of social and emotional competence which are: self‐awareness, social awareness, responsible decision making, self‐management and relationship skills. The columns of the EGM will represent outcomes. The size of the bubbles within each cell of the map will correspond to the number of studies represented. The colour of the bubbles will correspond to who facilitates or delivers the intervention. This is described in more detail below under ‘EGM framework’.

The visual, interactive EGM will be accompanied by a written narrative synthesis, describing the characteristics of the included studies and the number of included studies across each coding category of the map. A PRISMA^2^ flow diagram will also be included to describe the flow of studies through the search and screening process.

### 1.3 Conceptual framework of the EGM

#### 1.3.1 What is SEL?

**Figure 1:**
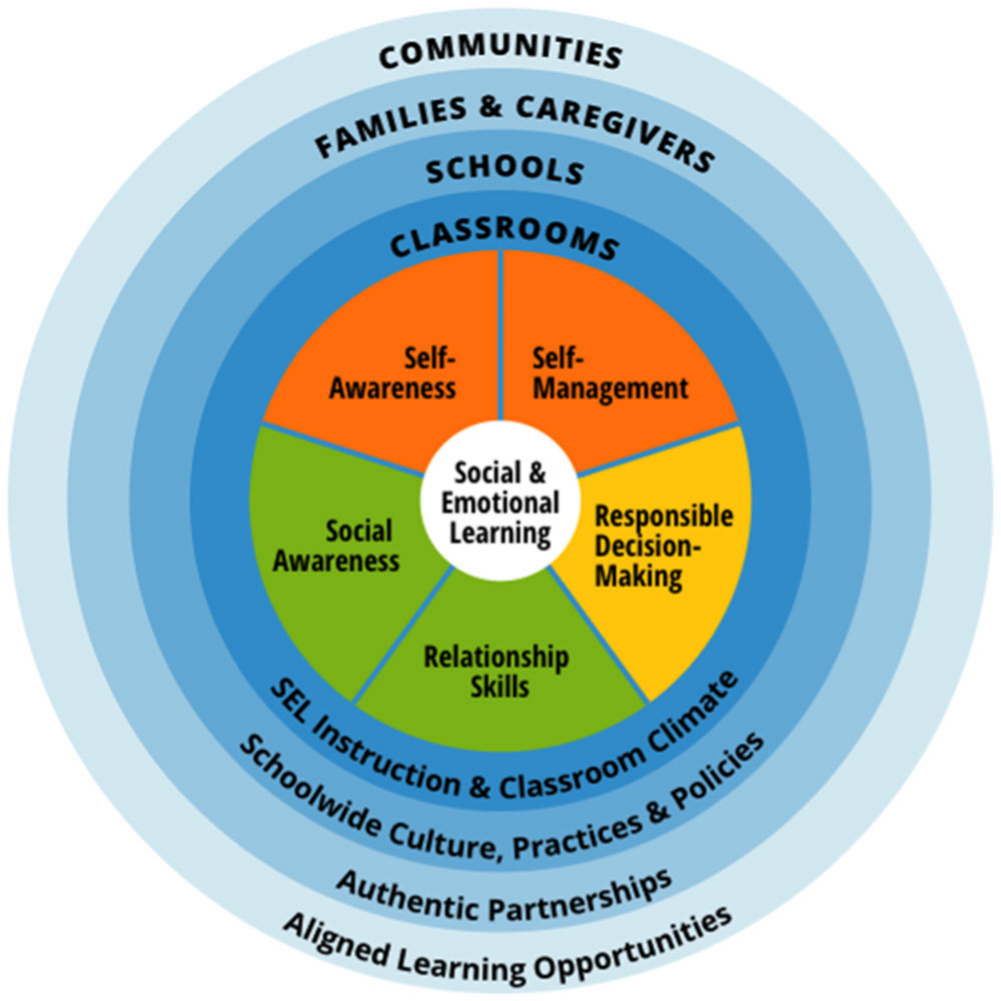
Casel framework. *Source*: https://casel.org/fundamentals-of-sel/what-is-the-casel-framework/.

SEL has been defined in different ways. Definitions tend to encompass a range of competencies, concepts and areas for development. For example, the American Collaborative for Academic, Social and Emotional Learning (CASEL, 2015) describe and defined the SEL framework as five core competencies: self‐awareness; self‐management; social awareness; relationship skills; and responsible decision making (Figure 1).

Waters and Sroufe (1983) also describe these social and emotional competencies as being important in enabling children ‘to generate and coordinate flexible, adaptive responses to demands and to generate and capitalize on opportunities in the environment’ (p80). In the UK, The Young Foundation (McNeil et al., 2012) have identified a core set of evidence‐based social and emotional capabilities shown to be important throughout the lifespan, including: communication; confidence and agency; planning and problem solving; relationships and leadership; creativity; resilience and determination; and managing feelings.

Cefai et al. (2018) defined social and emotional education as the development of competences in self‐awareness, self‐management, raise social awareness and improve the quality of social relationships. These competencies include children's capacity to understand themselves, express and regulate emotions, develop healthy and caring relationships, empathy, resolve conflict constructively, make good and constructive decisions as well as dealing with social and academic tasks.

Whilst core competencies vary in their definitions and scope between authors and agencies, a consistent pattern emerges demonstrating the array and reach of SEL and its related competencies.

As well as SEL definitions, there are several SEL frameworks available (e.g., WHO Skills for Health, UNICEF MENA Life Skills and Citizenship Education). CASEL was selected for this systematic review as it is an integrated framework involving educators, families, and communities to provide and integrated response to support and develop SEL. This is relevant as SEL occurs in a multilevel ecological system of contexts and relationships (Weissberg et al., 2015).

CASEL was developed as an evidence‐based SEL model supported by continuous research and evaluation (Weissberg et al., 2015). CASEL has also been integrated into preK‐12 education and is therefore relevant for this systematic review based on school‐based SEL interventions. This framework is widely used in several countries around the world (Brush et al., 2022).

#### 1.3.2 The importance of SEL

It has been evidenced both nationally and internationally (Barry et al., 2013; Weare & Nind, 2011; Yoshikawa et al., 2015) that improving SEL allows children to connect with others and to begin to learn in a more effective way, thereby increasing their chances of success both in school and in life. Many countries have endeavoured to embed SEL into school ethos and culture. For example; in England personal and social development is addressed at the policy and practice level through the inclusion of building character and resilience as a priority by English Department of Education's (Department of Education, 2019); In America, CASEL work to support the integration of SEL in education through research, practice and policy; In Australia, SEL has been embedded in the national curriculum under personal and social capability (Collie et al., 2017).

There is a growing consensus in academic and policy circles regarding the importance of children's social and emotional development and its links to behavioural and health outcomes (Ciarrochi et al., 2002; NICE, 2023; Petrides et al., 2004). Children who fail to achieve developmental milestones associated with SEL may be at risk of failing to make meaningful relationships with their peers and with the school situation (Zins & Elias, 2007; Zins, 2004). Social and emotional outcomes are related to educational outcomes (Duckworth & Seligman, 2005), emotional wellbeing (Eisenberg et al., 2010) and general life trajectories (Daly et al., 2015; Moffitt et al., 2011) and overall wellbeing (Taylor et al., 2017). Educationally, a range of social and emotional factors have been found to have an impact on educational achievement (Banerjee et al., 2014; Durlak et al., 2011), including willingness to learn, openness to new experiences and one's ability to interact with peers and teachers in the school situation. Corcoran et al. (2018) found that SEL had a positive effect on reading, maths and science attainment. Regarding mental health, research on early childhood development has shown that early experiences, including relationships with parents, caregivers, relatives, teachers and peers determine the foundation for sound mental health (Centre on the Developing Child Harvard University, 2023).

Given the increasing emphasis on SEL in both the educational research and policy arenas, there has been increased importance placed on SEL in schools and the need for facilitating optimal development of SE competence in every child. A variety of school‐based programmes have been developed which aim to target, remediate and/or improve social and emotional skills in children, from the very beginning of their educational careers. Within the context of education and schooling, these programmes are particularly important for several reasons. First, children do not learn in isolation, but rather construct meaning based on their life experiences (Vygotsky, 1980) and the relationships they have built can either facilitate or impede the learning process (Zachary, 2011; Zeidner et al., 2012). Second, social and emotional skills are necessary antecedents for learning and constructing knowledge and can help ensure that children are ready, willing and able to learn. Third, strong social and emotional skills enable children to work together, work with others and work alone in the school setting.

#### 1.3.3 Moderating factors

There is evidence to suggest that certain groups of children are more likely to have difficulties in specific areas of social and emotional development. Children from socially disadvantaged communities and children who suffer maltreatment, are found to be less likely to develop social and emotional skills in line with their peers (Blair & Raver, 2012). Research on similarities and differences between social and emotional development in boys and girls is equivocal with some studies suggesting girls and boys develop in different ways (Lehmann et al., 2013) while others conclude that these differences may be exaggerated (Else‐Quest et al., 2012). Finally, age is likely to be a moderating factor. While early intervention has been shown to produce greater impact, this is not the case for all relevant outcomes (Sklad et al., 2012). Understanding the developmental trajectory of social and emotional skills and identifying key developmental stages may help identify the age at which interventions are likely to confer most benefit.

#### 1.3.4 Description of the intervention

This EGM will focus on curriculum based^3^ SEL interventions delivered in preschool or primary/elementary schools aimed at improving social and emotional skills among pupils. It will include any universal programme, delivered on a whole‐class or school basis. The interventions’ primary goal must be to improve social and emotional competence and be delivered in a pre‐school/kindergarten or primary/elementary school setting as part of the normal school day.

The intervention must be structured, include a taught component and be delivered directly to children and involve the active participation of the child. The aims of the programme must be explicitly related to child gains, as opposed to those which focus solely on teacher competencies or school ethos. The intervention must also be a curriculum‐based social emotional learning program. Interventions may be delivered by the class teacher, other school personnel or non‐school personnel. Interventions must be delivered for a minimum of one school term.

#### 1.3.5 How the intervention might work

Whilst interventions are varied in terms of their scope and their delivery, they typically adopt either a preventative or remediative approach and may use a range of theoretical approaches, such as Bronfenbrenner's ecological theory or the achievement model of emotional literacy (Rivers & Brackett, 2010). Many SEL interventions in preschool and primary/elementary school are based upon an implicit or explicit understanding of how social and emotional skills develop among children at this age. Rarely, however, do interventions outline a logic model which clearly sets out how the intervention creates or facilitates change in the desired outcomes. To this end, most interventions encourage and help children recognise and identify emotions or undesirable behaviours. Children are then taught, usually through scene setting, discussion and/or modelling techniques, skills to help them reflect on the problem and think through (and thus modify) the various and alternative actions they might take when faced with a difficult or conflict‐related situation.

Jones et al. (2018) have identified common features of effective SEL programmes. These are (i) Programmes occur in contexts supportive of social and emotional development. (ii) Build adult competencies by promoting social and emotional competence of teachers. (iii) Build family, school and community partnerships that support children in the environments and contexts where children learn live and grow (iv) target developmentally appropriate skills including emotional processes, social/interpersonal skills, cognitive regulation and executive functions. (v) Short‐ and long‐term outcomes and goals that can be tracked over time on children's development and growth.

A range of programmes have emerged that target children who are not meeting the developmental milestones associated with SEL. Some of these milestones include, for example: being able to understand complex and simultaneous feelings; consider multiple perspectives; empathise or sympathise with others; and recognise, regulate and express emotions effectively to create and sustain personal and social relationships. In so doing, SEL interventions aim to support children to enhance their positive emotions and behaviours, whilst also moderating their negative emotions and associated behaviours.

Programmes such as *PATHS* (Promoting Alternative Thinking Strategies) are implemented widely in several countries and are designed to facilitate the development of self‐control, emotional awareness and interpersonal problem‐solving skills. PATHS does this by teaching children strategies to help them regulate their emotional response to a difficult situation by: stopping to reflect on the problem, thinking through alternative solutions and based on this, choosing an appropriate course of action. Here, the programme's aims, and objectives are closely tied with the developmental achievements which have been set out in previous literature. Similarly, *Roots of Empathy*—a classroom‐based SEL intervention ‐ seeks to promote prosocial behaviour and reduce aggressive behaviour by enhancing children's emotional recognition, empathy, and emotional regulation. It is a structured curriculum‐based programme that involves a mother and baby coming to class. Children observe the baby's development over the period of a school year and are taught to ‘recognise’ the baby's feelings and reflect on their own and others' feelings. Learning about the baby's development, how the baby might be feeling, why they might be upset or happy and observing the loving parent–child relationship provides children with a model of responsible parenting from which to learn. The intervention relies on a mixture of modelling, discussion, and storytelling to teach children about their own and others’ emotions and behaviours.

Based on the SEL framework suggested by CASEL, explicit instruction in SEL skills (provided through the intervention) combined with teacher instructional practices that are both integrated with the academic curriculum and school ethos, should lead to the acquisition of SEL skills, improved attitudes towards self, others and learning as well as an enhanced learning environment. In turn, these changes result in positive social behaviour, fewer conduct problems, lower emotional distress and improved academic performance.

For the purpose of the current EGM, the outcomes will include Prosocial behaviour; SEL skills (emotion regulation, emotion recognition, empathy, problem solving); attitudes towards self and others (self‐esteem, peer relationships, school enjoyment); emotional distress (anxiety, depression, social withdrawal); educational attainment; and any reported adverse effects.

Whilst certain background or demographic characteristics may moderate the impact of a programme on certain groups of children or families (previously discussed), other programme‐related factors—such as quality and fidelity of implementation—can also moderate effects. Several programme factors emerge which could be seen to be important when implementing a programme. As mentioned in Clarke et al. (2015) review, parental involvement may be an area which facilitates success of an intervention. Here it was found that when parents were involved and knowledgeable about the process being undertaken children were more likely to make significant gains. Additional factors which may lead to greater successes include teachers and other school staff implementing the programme compared to instructors from outside the school (Durlak et al., 2011, p. 13) which can allow children to make greater gains, perhaps because they feel calm and confident with the instructor. There is also some evidence to suggest that structured programmes which incorporate training for those delivering the program, are interactive in nature and which guide young people towards a specific set of goals are more likely to be successful (Smith et al., 2004). Moreover, there is some evidence to suggest that multi‐component programmes may have added benefits compared with single component programs (Adi et al., 2007; Catalano et al., 2004; Humphrey et al., 2010; Wells et al., 2003). Factors regarding programme design and implementation are also influential, with programmes which were identified as well‐designed and well implemented being the most impactful (Durlak et al., 2011). Stage of programme development may affect the success of the intervention, with efficacy trials conducted under controlled conditions and often led by the programme developer resulting in greater effects than effectiveness trials conducted under more ‘real world’ conditions (Wigelsworth et al., 2016).

These programme content and implementation factors will also be documented in the coding framework.

### 1.4 Why it is important to develop this EGM

The importance of SEL is now well recognised, and there has been significant growth in the number and type of SEL programmes offered in schools. Due to the scale of the existing evidence in SEL this EGM is a suitable option to map the evidence in a systematic format to identify and inform more targeted (future) reviews. The Campbell and Cochrane systematic review libraries were searched for completed and ongoing reviews relevant to this area. To the authors’ knowledge there are currently no other EGMs focused on SEL.

## 2 OBJECTIVES

The objective of this EGM is to identify and map all primary studies (including randomised and cluster randomised trials) and systematic reviews on universal, school‐based SEL programmes for young children (3–11 years) to create a live, searchable, and publicly available EGM.

## 3 METHODOLOGY

This EGM will follow the Campbell Collaboration EGM guidelines (White et al., 2020). These guidelines consist of six phases:

1) Scoping and development of the EGM framework
2) Systematic and comprehensive searches
3) Screen for eligibility (title, abstract, full text)
4) Data extraction
5) High‐level appraisal of systematic reviews
6) Analysis (applied inclusion and exclusion criteria)

### 3.1 Defining EGMs

EGMs are defined as systematic evidence synthesis products which identify and display available evidence corresponding to a specific research question (White et al., 2020). Utilising the same robust searching strategies as systematic reviews, but extracting fewer data from each included study, EGMs allow researchers to accurately estimate the shape and size of an evidence base, without extracting or synthesising the outcome data reported within the included studies. EGMs clearly identify the areas that are ripe for systematic review and highlight the evident gaps, where more primary research is needed, thereby minimising research waste and increasing the discoverability of relevant research.

The benefits of EGMs are several:

1) Researchers and funders can target the areas where knowledge gaps exist and concentrate their efforts and research on generating much needed and valuable knowledge.
2) Maps can be useful tools to inform important policy and practice decision making.
3) Minimise research waste and duplication of already existing knowledge.
4) Members of the public can have access to relevant and evidence‐based information on the topic of SEL.

### 3.2 EGM framework

The framework of the proposed EGM is based on the conceptual framing of the originally intended review. It will follow the standard matrix format. The columns will represent the outcomes of the SEL intervention which include primary domains (in bold) and secondary domains (in parentheses):

- **Behaviour** (prosocial behaviour and conduct problems)
- **SEL skills** (emotion regulation, emotion recognition, empathy, problem solving)
- **Attitudes towards self and others** (self‐esteem, peer relationships, school enjoyment)
- **Emotional distress** (anxiety, depression, social withdrawal)
- **Educational attainment**
- **Adverse effects**

The rows of the matrix will represent different types of interventions, categorised according to the CASEL framework which identifies five interrelated areas of social and emotional competence (https://casel.org/sel-framework):

1. Self‐awareness
2. Social awareness
3. Responsible decision making
4. Self‐management
5. Relationship skills

The size of the bubbles within each cell of the map will correspond to the number of studies represented. The colour of the bubbles will correspond to the intervention facilitator (i.e., whether the intervention is delivered by the teacher, an external facilitator or other/multiple school personnel).

In addition, it will be possible to filter the map according to:

- Age (and/or grade) of the target population
- Level of intervention (e.g., standalone, or integrated curriculum, classroom focused change, teacher training, school level, school systems change)
- Type of study comparison group (e.g., waitlist, treatment as usual, another active, or inactive intervention)
- Whether the programme is manualised or not
- Country in which the study was conducted.

The visual, interactive EGM will be accompanied by a written narrative synthesis which will describe the characteristics of the included studies, as well as reporting the number of included studies across each coding category of the map. A PRISMA flow diagram will describe the flow of studies through the search and screening process.

### 3.3 Criteria for including and excluding studies

#### 3.3.1 Characteristics of the relevant studies

Given the broad definition of SEL and the wide variety of school‐based interventions that now exist, studies will be varied in terms of their design, methodology, implementation, outcomes and measurement.

Four representative studies that could potentially meet the inclusion criteria for the current EGM, are detailed below.

1. Domitrovich et al. (2007) report a cluster randomised controlled trial evaluation of the PATHS (Promoting Alternative Thinking Strategies) curriculum (Kusché & Greenberg, 1994). This classroom‐based curriculum is designed to reduce problem behaviour whilst promoting social competence. This waiting list control trial involved twenty classrooms in Pennsylvania, ten randomly allocated to each of the control and intervention groups. The intervention was delivered to children of nursery age (between 3 and 4) by a trained class teacher, who implemented weekly lessons (30) and extension activities across a 9‐month period.
2. Snyder et al. (2012) conducted a randomised controlled trial on Positive Action, a character development programme which aims to improve school quality, improve academics, student behaviour, and character and to mitigate problem behaviours. This universal, school wide programme includes a school‐wide climate development component, teacher/staff training, a coordinator's manual, school counsellor's programme, and PA coordinator/committee guide; and family‐ and community‐involvement programmes. The sequenced elementary curriculum consists of six units which are divided into 140, 15‐ to 20‐min lessons per grade, per academic year, provided by classroom teachers. This trial involved 20 racially/ethnically diverse schools and was conducted between 2002 and 2006. Teacher, parent and student data were collected in pre‐ and post‐tests and additional, school‐level archival data were used to examine programme effects at 1‐year post‐ trial.
3. Mendelson et al. (2010) conducted a randomised trial assessing the feasibility, acceptability, and preliminary outcomes of a pilot school‐based mindfulness and yoga intervention. An existing intervention was manualised and structured into sessions which ran for 4 days a week for 12 weeks. Two schools took part, with half of the children in each school being assigned to the intervention group, which consisted of around 50 children.
4. Holen and colleagues (Holen et al., 2012) evaluated Zippy's Friends, a universal school‐based programme that aims at strengthening children's coping skills, with 1,483 children in 35 schools aged between 7 and 8. This manualised, structured programme is delivered in 24 weekly lessons and is based on six stories about three cartoon characters. The main objective of the programme is to prevent psychological problems by increasing children's coping repertoire and giving them various ways of coping with problems.

#### 3.3.2 Criteria for including and excluding studies

##### Types of study designs

Primary studies including randomised controlled trials or cluster randomised controlled trials will be included, as well as systematic reviews on universal, school‐based SEL programmes for young children to create a live, searchable, and publicly available EGM.

We anticipate many included studies and so can reasonably restrict our analysis to only the highest quality research designs. This highlighted the need for targeted and more relevant systematic reviews in the future.

##### Types of participants

Children attending preschool or primary/elementary schools. This population is typically between the ages of 3 and 11 years.

Where a study includes participants from both preschool or primary schools and secondary schools, reasonable attempts will be made to extract or source the data for only the children attending preschool/primary/elementary school from published and unpublished documentation. If data us unavailable authors will be contacted to request descriptive details for the participants of interest.

Studies which are in special schools, or which focus on children with identified special educational needs or social, emotional or behavioural difficulties will be excluded.

##### Types of interventions

Studies will be included if the intervention is a universal, school or classroom‐based programme which is delivered, via a set curriculum, to a whole class or whole school and which primarily aims to improve social and emotional competencies of children. The intervention must be a curriculum‐based social emotional learning programme.

The intervention must be delivered directly to children and involve the active participation of the child. The aims of the programme must be explicitly related to child gains, as opposed to those which focus solely on teacher competencies or school ethos.

Interventions may be delivered by the class teacher, other school personnel or non‐school personnel. Interventions must be delivered for a minimum of one school term.

Studies must include an inactive comparison condition that could include:

- **No treatment**.
- **Treatment as usual** where pupils receive their normal level of support or intervention. Details of what this consists of will be extracted.
- **Waiting list** where schools or classrooms are randomly assigned to receive the intervention later. Details of what happens to waitlisted participants will be extracted.
- **Attention control**, where participants receive some contact from researchers but both participants and researchers are aware that this is not an active intervention.
- **Placebo** where participants perceive that they are receiving an active intervention, but the researchers regard the treatment as inactive.

Studies with an inactive control compared to two or more intervention arms can be included and the sample size of the control group will be divided by the number of eligible intervention arms to avoid double counting control group participants in any meta‐analysis.

The following types of studies will be excluded:

- Studies in which the SEL programme was delivered in an after‐school setting or without a school‐based component (e.g., in youth clubs, summer clubs, sports or social clubs, or through parenting groups).
- Studies in which the SEL programme targets specific groups associated with the outcomes of interest (e.g., pupils with special educational needs); and
- Studies in which the programme is not delivered directly to children (e.g., interventions aimed at changing the behaviour or attitudes in teachers without any direct involvement of children).
- Studies in which the programme is not delivered according to a pre‐specified curriculum or manual (e.g., school providing leisure facilities to pupils).
- Studies in which the programme is delivered as a one off or infrequent intervention.

Kratochwill et al. (2009) represent an example of a study that is likely to be excluded. This study evaluated the Families and School Together (FAST) programme (McDonald et al., 1997). Here, children were recruited from schools and the programme delivered in the school setting. The programme aimed to improve several outcomes, including parental involvement, child behaviour and teacher perceptions of attainment. To do this it delivered eight weekly sessions where children and parents took part in games and activities together. Whilst this study does include some aspects of social and emotional development, the focus was on parents who are working alongside their children. This is a multi‐family, group intervention, rather than one which focusses on the child and their social and emotional development. As there is no direct taught element which is delivered to the child this study would not meet the inclusion criteria.

### 3.4 Search strategy

To find all eligible studies, the following data sources will be searched and consulted. This comprehensive search will include multiple electronic databases, research registers, grey literature sources and reference lists of reviews and relevant studies. We will not restrict the study selection in terms of language, date or publication studies. We will not restrict the study selection in terms of language, date or publication status. Each of the following databases and trial registries will be searched using the search strings set out in Supporting Information: Appendix 1:

a) British Education Index
b) Education Abstracts (EBSCO)
c) ERIC
d) MEDLINE
e) PsycINFO
f) Web of Science/Knowledge Database Science Citation Index
g) Web of Science/Knowledge Database Social Science Citation Index
h) ClinicalTrials.gov
i) National Registry of Evidence‐based Programs and Practices Relevant reviews will be searched for in the following databases:
j) Database of Abstracts of Reviews of Effectiveness
k) The Campbell Library
l) Cochrane Collaboration Library
m) Evidence for Policy Practice Information and Coordinating Centre (EPPI‐Centre) We will also search grey literature and the following databases and websites will be used:
n) CASEL
o) Education Endowment Foundation
p) Google Scholar—using a series of searches and screening the first two pages of results for each search.
q) ProQuest Dissertations and Theses
r) WorldCAT
s) OECD Education Library
t) Opengrey
u) World Health Organization

Reference lists of relevant reviews and included studies will be screened and forward citation searching of included studies will be carried out through Google Scholar. These web searches will be limited to the first two pages of search results. Prominent authors in the field will also be contacted. In addition, a final step towards the end of analysis, a manual search of the most recent issue(s) of key journals will be conducted. To do this, the top 10 journals that have provided included studies so far, will be identified and their most recent issues will be checked.

One reviewer will conduct the database searches, remove duplicates and obviously irrelevant records. We anticipate that the searches will result in a very large number of records to screen and so to ensure robustness, each report will be screened by title and abstract by two reviewers. Potentially eligible studies will then be retrieved in full text form and two reviewers will screen each full text. Any disagreements will be discussed with the wider review team until a consensus is reached.

#### 3.4.1 Search terms and keywords

The search strategy has been developed using a modified version of the pearl harvesting approach. First, one author (JR) extracted keywords from 10 randomly selected relevant studies. Each of the terms extracted were then searched individually within the thesauri in ERIC, British Education Index and Psycinfo and all additional terms added to the list of possible search terms relating to either: the participants; intervention setting; study design; or outcomes of interest. All authors then suggested additional terms that had not been identified (e.g., nursery school did not appear in any article of thesauri). Two authors (JR and JH) then screened the full list of terms and removed any duplicate, overlapping or irrelevant terms. The search string samples below therefore were generated by a combination of terms originating from the literature, terms originating from the review team and terms originating from a thesauri search.

A combination of four search strings will be used relating to: (1) participants; (2) the intervention setting; (3) the study design; and (4) the outcomes of interest. A sample search strategy is provided in Supporting Information: Appendix 1. Search strings and search limits will be modified to be suitable for each database. Search for exact phrases or proximity searching will be used to increase search specificity. In addition, searches for a number of known named interventions will also be undertaken (see Supporting Information: Appendix 1).

#### 3.4.2 Description of methods used in primary research

Primary studies including randomised controlled trials or cluster randomised controlled trials will be included, as well as systematic reviews on universal, school‐based SEL programmes for young children to create a live, searchable, and publicly available EGM.

#### 3.4.3 Details of study coding categories

A coding framework has been devised and piloted (see Supporting Information: Appendix 2). Coding will be carried out by trained researchers and each study will be coded by two members of the review team independently. Discrepancies will be discussed, and a consensus agreed. The coding categories include:

- Study design (review or trial)
- Country in which the study was conducted.
- Name of the intervention if applicable
- Comparison group (wait list, treatment as usual, another inactive intervention, another active intervention)
- Intervention characteristics (as described by the CASEL framework)
- Intervention provided by (e.g., teachers, external facilitator, other school staff…)
- Level of intervention (e.g., standalone curriculum, integrated curriculum, classroom focussed change.)
- Outcomes (as described above)
- Follow‐up (whether and when follow up data were collected)
- Implementation (whether the intervention is manualised and was implementation assessed in the study)

#### 3.4.4 Risk of bias

The methodological quality of systematic reviews will be assessed in duplicate using AMSTAR 2 (Shea et al., 2017). Discrepancies will be discussed, and a consensus agreed. The methodological quality of primary studies will not be assessed.

## 4 ANALYSIS AND PRESENTATION

### 4.1 Unit of analysis

Each report included in this EGM is either a systematic review, an overview of systematic reviews or a primary study. Where there are multiple reports of a single study, these will be considered as a single study. Where a single report includes multiple studies, these will be represented in the map separately.

### 4.2 Planned analyses

The visual EGM will be developed by using the EPPI‐Mapper. This is a tool for visualising maps of research evidence. The tool uses an exported file from EPPI‐Reviewer to create a map that can be accessed from any web browser.

The visual, interactive map will be supplemented by a written narrative synthesis which will describe the characteristics of the included studies, as well as reporting the number of included studies across each coding category of the map. This narrative synthesis will also discuss the potential uses of this EGM for different sectors. The implications of the findings and the limitations of the EGM will be discussed in detail in this section.

Additionally, and to aid transparency, a PRISMA flow diagram will describe the flow of studies through the search and screening process of all interventions included in the EGM.

### 4.3 Presentation

As described in more detail in Section 3.2 EGM Framework, this EGM will follow the standard matrix format. The columns will represent the outcomes of the SEL intervention (e.g., prosocial behaviour, SEL skills, attitudes, emotional distress, etc). The rows of the matrix will represent different types of interventions, categorised according to the CASEL framework (e.g., self‐awareness, social awareness, etc.). The size of the bubbles corresponds to the number of studies represented. The colour of the bubbles corresponds to the intervention facilitator (i.e., teacher, an external facilitator or other/multiple school personnel).

## ADVISORY GROUP

Members of the current Review Team (Connolly, Miller and Sloan) have been part of a team that has undertaken a large cluster‐randomised controlled trial of the Roots of Empathy social and emotional learning programme in Northern Ireland. This trial has been funded by the National Institute for Health Research (ISRCTN07540423). A Roots of Empathy Regional Planning Group has been established to oversee the delivery of the programme.

The Group is led by the Public Health Agency and includes representatives from each of the five Health and Social Care Trusts in Northern Ireland charged with the delivery of the programme.

## ROLES AND RESPONSIBILITIES

- Content: Connolly, Hanratty, Miller, Roberts, Sloan.
- EGM methods: Hanratty, Miller.
- Information retrieval: Miller, Rodriguez, Hanratty, Roberts, Sloan, Brennan‐Wilson, Bradshaw, Coughlan, Gleghorne, Dunne, Millen, Smith, O'Sullivan.
- Screening and data extraction: Miller, Rodriguez, Hanratty, Roberts, Sloan, Brennan‐Wilson, Bradshaw, Coughlan, Gleghorne, Dunne, Millen, Smith, O'Sullivan.
- Analysis and reporting: Miller, Rodriguez, Hanratty

## POTENTIAL CONFLICTS OF INTEREST

None of the review authors has a financial interest in this review. None of them have been involved in the development of interventions to improve social and emotional learning. Some of the authors (Connolly, Miller, Hanratty, Sloan) have either completed and/or are currently running trials of interventions that may fall within the scope of this present review. Connolly is currently supervising a PhD student whose doctoral research involves the development and pilot evaluation of a preschool social and emotional learning programme that may fall within the scope of this present review. Hanratty is currently conducting a Cochrane systematic review of child‐focused interventions for anger and aggression in young children.

## PRELIMINARY TIMEFRAME

Date you plan to submit a draft EGM: 30 June 2022.

## PLANS FOR UPDATING THE REVIEW

Review authors will update the EGM 3 years after the submission date.

## Supporting information

Supporting information.

## Footnotes

1 CASEL stands for: American Collaborative for Academic, Social and Emotional Learning.

2 PRISMA Flow diagrams summarise the screening process. It records the initial number of articles and describes the different stages of the selection process.

3 Curriculum based referred to being part of the internal school policy. The manual refers to the concrete, written version of the intervention, which may or may not be included in the school curriculum.
