# Supplementary material for: UPDATED PROTOCOL: Universal school‐based programmes for improving social and emotional outcomes in children aged 3–11 years: An evidence and gap map
Source: Campbell Syst Rev. 2023 Aug 4;19(3):e1346. doi: 10.1002/cl2.1346 (PMC10400952; doi:10.1002/cl2.1346)
Supplement: Supplementary file 1 — Supporting information. [file CL2-19-e1346-s001.docx]

**Appendix 1: Search strings**

Searched in all text fields unless otherwise specified.

# Participants

In order to identify studies that concern children in a school setting we combine broad terms for age with (school* or class or classroom). This is to improve the precision of the search and avoid returning too many irrelevant records concerning older children, or papers concerning other schools settings, for example medical school, law school. The search strings for participants therefore come in two parts;

(Child* or youth* or boy* or girl* or Young children or Young people or preadolescen* or pre-adolescen* or early adolescen* or earlyadolescen* or Junior infant* or senior infant* or "reception class" or First Class or Second Class or Third Class or Fourth Class or Fifth Class or Sixth Class or Grade* 1 or Grade* 2 or Grade* 3 or Grade* 4 or Grade* 5 or Grade* 6 or Intermediate grades or Grade* one or Grade* two or Grade* three or Grade* four or Grade* five or Grade* six or First Grade or Second Grade or Third Grade or Fourth Grade or Fifth

Grade or Sixth Grade or 1st grade or 2nd grade or 3rd Grade or 4th Grade or 5th grade or 6th Grade or Age* 3 or 3 year* old* or Age* 4 or 4 year* old* or Age* 5 or 5 year* old* or Age* 6 or 6 year* old* or Age* 7 or 7 year* old* or Age* 8 or 8 year* old* or Age* 9 or 9 year* old* or Age* 10 or 10 year* old* or Age* 11 or 11 year* old*) AND school* or class or classroom

OR

prekindergar* or pre-kindergar* or kindergar* or pre-k or pre-school* or preschool* or early childhood education* or (Nursery* adj1 (class* or school* or student* or pupil* or education)) or (elementary adj1 (class* or school* or student* or pupil* or education)) or (primary adj1 (class* or school* or student* or pupil* or education))

# Study design

(singl* or doubl* or trebl* or tripl*) adj3 (blind* or mask*) ) OR

Allocation or allocated or assigned or Causal Design or Cluster Design or Comparison Condition or Comparison group or Control condition or Control Group or Control groups or Controlled trial or cluster trial or random* or RCT or clinical trial OR

((evaluat* or prospective*) adj3 (study or studies)) OR ((Process Evaluation or Program* evaluation)) OR

((Treatment Condition or Treatment effectiveness evaluation or Treatment group or Treatment outcomes))

# Social-emotional terms

Terms presented alphabetically on separate lines for clarity, all terms combined with “OR” (Acting out)

(adjustment adj1 (disorder* or emotion* or school)) (aggression or aggressive behavio* or anger) (attachment adj1 (problem* or disorder*))

(behavio* adj1 (anti-social or antisocial or development or difficult* or management or problem* or high risk or risky or social or prosocial or pro-social or helping))

(bully*)

(cognitive function or conflict management or conflict resolution or coping or delinquen*) (communicat* adj1 (skill* or problem* or interpersonal))

(emotion* adj1 (control or regulat*))

(emotional intelligence or emotional wellbeing or managing feelings) (empathy or executive function or hostility or impulsiv*)

(interpersonal adj1 (competence or conflict or interaction or relation* or skill*)) (mental health)

(mindfulness)

(non-cognitive skill)

(peer adj1 (relation* or acceptance or rejection or pressure))

(perspective taking or positive youth development or pro?social or problem solving or relationship skill*)

(resilience or resiliency) or grit or character development or 21st century skill

(self adj1 (control or inhibition or awareness or efficacy or esteem or perception or regulat* or respect))

(social adj1 (adjust* or alienat* or emotion* or attitud* or cognition or competenc* or development or interaction or isolation or learn* or participation or problem* or skill*)) (social and emotional learning) or socio-emotional learning

(stress adj1 (manag* or reduc* or control)) (violence or violent)

# Additional limits

Due to the very large number of records (>13,000) returned when testing the searches in PsycInfo, additional limits were added by combining the strings above AND the following; "Curriculum & Programs & Teaching Methods”.cc or (intervention* or program* or course* or polic* or practice* or curricul* or environment* or prevent* or training or treat* or school-based or school based or class based or classroom based or class-based or classroom- based or universal)

# Known interventions

Finally, we added a search for the following known interventions;

“Roots of Empathy” OR PATHS OR “promoting alternative thinking strategies” OR “Skills for Life” OR “The Good Behavio* Game” OR “Bounce Back” OR “Zippys Friends” OR “RTime” OR “Circle Time” OR “Primary SEAL” OR “Positive Action” OR “Incredible Years” OR “Tools of mind” OR “Head Start REDI” or “incredible years”.

For all strings proximity searching will be employed using notation appropriate for each database (e.g. “primary adj1 school”, “fourth N1 grade” to limit the number of irrelevant records retrieved.

**Appendix 2: Data extraction framework**

| **Study Characteristics** |  |  |
| --- | --- | --- |
| Review or Trial | Systematic Review | **Checkbox** |
|  | RCT |  |
| Age | Early Childhood Education | **Checkbox** |
|  | Preschool/nursery 3-4 |  |
|  | Pre-K/Reception/P1 age 4-5 |  |
|  | Kindergarten/KS1 year 1/P2 age 5-6 |  |
|  | 1^st^ Grade/KS1 Year 2/ age 6-7 |  |
|  | 2^nd^ Grade/ KS1 Year 3/ 7-8 years |  |
|  | 3^rd^ Grade/ KS1 Years 4/ 8-9 years |  |
|  | 4^th^ Grade /KS1 Year 5/9-10 years |  |
|  | 5^th^ Grade/KS1 Year 6/10- 11 years |  |
| Country | Austria  Australia  Belgium  Belize  Brazil  Canada  China  Chile  Croatia  Democratic Republic of Congo  Denmark  Egypt  Finland  Germany  Greece  Holland  Honduras  Hong Kong  India  Indonesia  Iran  Ireland  Israel  Italy  Jamaica  Japan  Lebanon  Luxemburg  Malta  Mauritius  Mexico  Nepal  Netherlands  New Zealand  Nigeria  Norway  Palestine  Poland  Portugal  Scotland  South Africa  Spain  Sri Lanka  Sweden  Switzerland  Turkey  Uganda  United Kingdom  Venezuela  United States of America  Vietnam  Zambia  Global | **Checkbox** |
| **Intervention Information** |  |  |
| Named Intervention |  | **Free text** |
| Comparison | Wait-list | **Checkbox** |
|  | Treatment as usual | **Checkbox** |
|  | Another inactive intervention | **Checkbox** |
|  | Another active intervention | **Checkbox** |
| Intervention: Inputs/Targets/Behaviours | Self-awareness | **Checkbox** |
|  | Social awareness | **Checkbox** |
|  | Responsible decision making | **Checkbox** |
|  | Self-management | **Checkbox** |
|  | Relationship skills | **Checkbox** |
|  | Other (please specify) | **Checkbox** |
| Intervention provided by | Teachers | **Checkbox** |
|  | External facilitator | **Checkbox** |
|  | Other school staff | **Checkbox** |
|  | Multiple school personnel | **Checkbox** |
|  | Other | **Checkbox** |
| Level of Intervention | Stand alone curriculum for SEL skill development | **Checkbox** |
|  | Integrated curriculum for SEL skill development | **Checkbox** |
|  | Classroom focused change | **Checkbox** |
|  | Teacher training/ behaviour change | **Checkbox** |
|  | School level change | **Checkbox** |
|  | School systems/ structural change which restructures the school format or policies in order to enhance students´ social and emotional development | **Checkbox** |
|  | Parent involvement | **Checkbox** |
|  | School community partnerships | **Checkbox** |
| Outcomes* | Social and emotional skill performance | **Checkbox** |
|  | Attitudes | **Checkbox** |
|  | Positive social behaviour | **Checkbox** |
|  | Externalizing behaviour | **Checkbox** |
|  | Emotional distress | **Checkbox** |
|  | Academic attainment/ achievement | **Checkbox** |
|  | Cognitive skills/ executive function | **Checkbox** |
|  | Adverse effects not falling into any above category | **Checkbox** |
|  | Self-awareness | **Checkbox** |
|  | Social awareness | **Checkbox** |
|  | Responsible decision making | **Checkbox** |
|  | Self-management | **Checkbox** |
|  | Relationship skills | **Checkbox** |
|  | Mental health | **Checkbox** |
|  | None of the above | **Checkbox** |
|  | Other | **Checkbox** |
| Follow up | Baseline- prior to intervention | **Checkbox** |
|  | Immediate post test up to 1 month | **Checkbox** |
|  | Short term follow up (1-3 months) | **Checkbox** |
|  | Moderate follow up (3-6 months) | **Checkbox** |
|  | Extended follow up (6-9 months) | **Checkbox** |
|  | Further extended follow up (9-12 months) | **Checkbox** |
|  | Long term outcomes (1-2 years) | **Checkbox** |
|  | Follow up 2+ years | **Checkbox** |
|  | Other | **Checkbox** |
| Implementation | Manualized | **Checkbox** |
|  | Manual- unclear | **Checkbox** |
|  | Not manualised | **Checkbox** |
|  | Implementation assessed | **Checkbox** |
|  | Implementation unclear | **Checkbox** |
|  | Implementation not assessed | **Checkbox** |

*We have a comprehensive definition of SEL outcomes based on the CASEL framework. These categories are as follows:

SEL Outcomes:

1 = Social and Emotional Skill Performance includes performance assessments that measure a child’s personal, social, cognitive and affective skill performance derived from test situations or structured tasks, or in some cases during interviews assessing skills. Specific ratings of skill performance or social and emotional skills can come from the child, teacher, parent or independent observer ratings.  All ratings must reflect the demonstration of specific skills in a practice context (e.g., An observer rating of a child naming their emotions in response to a hypothetical situation is included in this category, but a general teacher rating stating that a child is able to control their emotions well would be in the Positive Social Behaviour category.)

2 = Attitudes includes only self-reported measures of self-perceptions, school bonding, and prosocial norms (i.e., conventional beliefs). Self-perceptions include both generalized measures of self-esteem, self-efficacy as well as more targeted measures such as academic self-concept. Prosocial norms include attitudes and beliefs towards several topics such as social justice, violence. School bonding includes measures of students’ feelings towards school and teachers, commitment to school, and perceptions of classroom environment.

4 = Positive Social Behaviour includes outcomes such as getting along with others, good social skills, and social adjustment taken from child, teacher, and independent observer.  These outcomes reflect daily behaviour rather than performance on hypothetical situations which would be placed into Social and Emotional Skill Performance.

5 = Externalizing Behaviour includes child, teacher, or independent observer ratings of disruptive school behaviour such as teacher reports of acting out in the classroom, outcomes that reflect behaviour problems and misconduct that occur outside of the school setting such as parent reports of problem behaviour, and measures of violence, aggression, and bullying which reflect naturalistic behaviours such as self-reports of violent acts. School record data of suspension and expulsion incidents are all included. Role play behaviours, behavioural intentions or attitudes towards violence should be placed in other categories.

6 =Emotional distress includes outcomes such as depression, anxiety, school stress, and social withdrawal.  These measures can be taken from child, teacher, or independent observer ratings.

7 = Academic Achievement includes both achievement tests which are measures of standardized reading and math achievements tests such as SAT and the ITB and grades which includes measures of students’ overall GPA or grades from a specific subject. Only outcomes taken from school records are included.

8 = Adverse effects not falling into any above category

9=None of the above
